# Identification of the PDI-Family Member ERp90 as an Interaction Partner of ERFAD

**DOI:** 10.1371/journal.pone.0017037

**Published:** 2011-02-16

**Authors:** Jan Riemer, Henning G. Hansen, Christian Appenzeller-Herzog, Linda Johansson, Lars Ellgaard

**Affiliations:** Department of Biology, University of Copenhagen, Copenhagen, Denmark; St. Georges University of London, United Kingdom

## Abstract

In the endoplasmic reticulum (ER), members of the protein disulfide isomerase (PDI) family perform critical functions during protein maturation. Herein, we identify the previously uncharacterized PDI-family member ERp90. In cultured human cells, we find ERp90 to be a soluble ER-luminal glycoprotein that comprises five potential thioredoxin (Trx)-like domains. Mature ERp90 contains 10 cysteine residues, of which at least some form intramolecular disulfides. While none of the Trx domains contain a canonical Cys-Xaa-Xaa-Cys active-site motif, other conserved cysteines could endow the protein with redox activity. Importantly, we show that ERp90 co-immunoprecipitates with ERFAD, a flavoprotein involved in ER-associated degradation (ERAD), through what is most likely a direct interaction. We propose that the function of ERp90 is related to substrate recruitment or delivery to the ERAD retrotranslocation machinery by ERFAD.

## Introduction

In the endoplasmic reticulum (ER), critical protein maturation steps including N-glycosylation and disulfide-bond formation take place. Upon folding, native proteins can exit the ER by the secretory pathway, whereas misfolded proteins and incompletely assembled protein complexes are generally retained by a protein quality control machinery [1]. Terminally misfolded proteins are degraded by the ER-associated degradation (ERAD) pathway, which involves the retrotranslocation of protein substrates to the cytosol and proteasomal degradation [2]–[5]. To alleviate unfolding, ERAD substrates that contain disulfide bonds might also have to be reduced before retrotranslocation [6], [7]. Recently, ERdj5, a member of the protein disulfide isomerase (PDI) family, has been demonstrated to facilitate this reduction step for certain ERAD substrates [8]. The electron donor for ERdj5 remains to be identified, and neither is it clear whether ERdj5 is the only reducing PDI-family member involved in ERAD.

The PDI family encompasses around twenty members in mammalian cells [9], [10]. These proteins perform functions in oxidative folding, protein retention, as chaperones and in ERAD. All PDI-family members contain one or more domains with a thioredoxin (Trx) fold. This fold typically consists of an N-terminal βαβ motif, a connecting loop containing one α-helix, and a C-terminal ββα motif. The β-strands form a central β-sheet that is surrounded by the α-helices. Redox-active PDI-family members contain one or more so-called **a** type Trx-like domains with an active site CXXC motif (C: Cys, X: any amino acid) that localizes to the N-terminus of the second α-helix [11]. The non-catalytic **b** type Trx-like domains lack the active-site cysteines, but instead provide important functionalities in substrate binding and/or chaperone activity [12]–[14].

We recently identified the ER-luminal flavoprotein ERFAD (ER flavoprotein associated with degradation; also known as FOXRED2) that participates in ERAD steps before retrotranslocation from the ER, although the precise mechanism of action remains unresolved [15]. ERFAD has also been shown to be upregulated by amyloid β in rat cortical neurons and SH-SY5Y cells [16]. This upregulation correlated with amyloid β neurotoxicity, the inhibition of proteasome activity and induced ER stress-mediated cell death [16]. ERFAD comprises consensus motifs for binding of the two redox cofactors flavin adenine dinucleotide and nicotinamide adenine dinucleotide phosphate. When purified from human cells the protein binds flavin adenine dinucleotide, and we have proposed a potential redox-function of the protein in ERAD [15]. In cells, ERFAD interacts with the ERAD factors OS-9, ERdj5, and SEL1L [15]. Here, we identify and characterize the previously unknown PDI-family member ERp90 as a further interaction partner of ERFAD.

## Results

### Co-immunoprecipitation with ERFAD identifies ERp90

**Figure 1:**
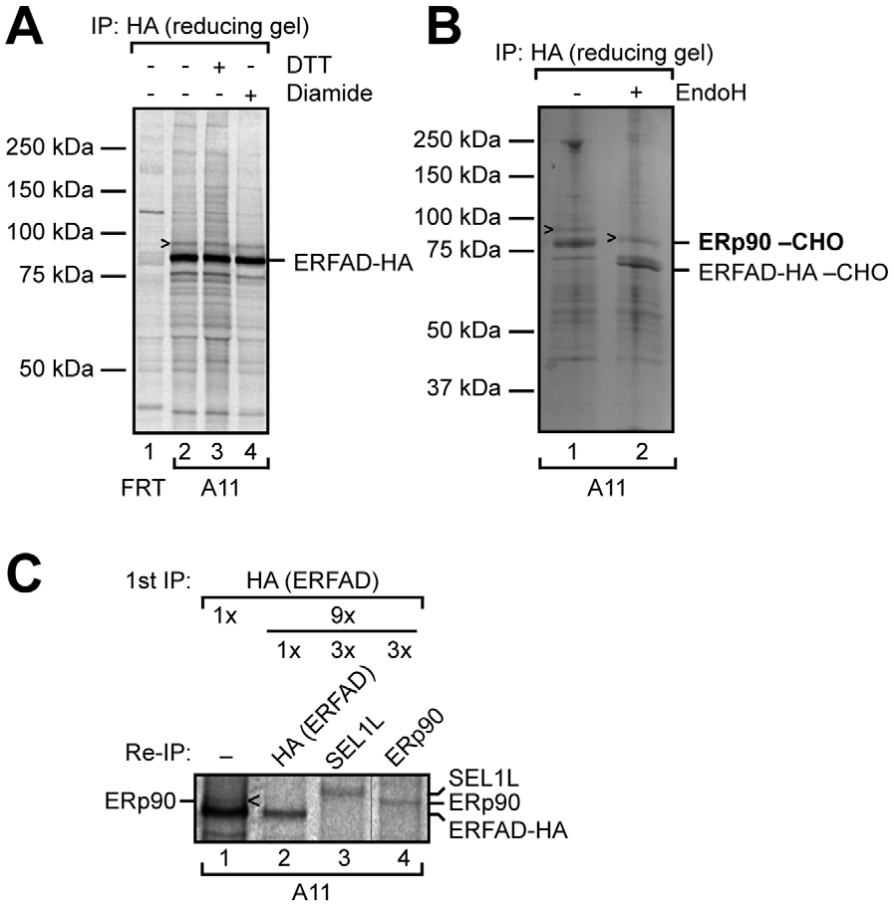
Identification of ERp90 as novel protein interacting with ERFAD-HA. (A) Immunoprecipitation of ERFAD-HA. Cells stably expressing ERFAD-HA (A11) and control cells (FRT) not overexpressing the protein were [^35^S] pulse-labeled for 16 hours, treated as indicated, Triton X-100 lysates immunoprecipitated with anti-HA, and samples separated by reducing SDS-PAGE. CHO, N-glycans. The arrowhead indicates the novel protein that was coimmunoprecipitated with ERFAD-HA. (B) The approach from (A) was upscaled and analysed by mass spectrometry. The positions of ERFAD-HA as well as of the identified protein – ERp90 – are indicated. (C) Immunoprecipitations on lysates of A11 cells that were [^35^S] pulse-labeled to steady state were performed with anti-HA. The immunoprecipitate was either analyzed directly (lane 1) or reimmunoprecipitated using HA- (lane 2), SEL1L- (lane 3) or ERp90- (lane 4) antibodies. The positions of the individual proteins are indicated and the glycosylated 90 kDa band coprecipitating with ERFAD-HA can thereby be attributed to ERp90. The hairline indicates that one lane has been removed.

We recently found ERFAD to interact with a number of well-known ERAD components and several other proteins in a complex that could be stabilized by crosslinking [15]. To further characterize this protein complex we sought to identify additional components. To this end, we used a HEK293-derived cell line stably expressing ERFAD with an HA tag inserted immediately prior to the C-terminal KEEL sequence (A11 cells, [15]). From a [^35^S]-methionine-labeled extract of these cells we immunoprecipitated ERFAD-HA (Figure 1A). The experiment revealed one clear candidate interacting protein that was not recovered from control cell lysates. This protein had an apparent size of ∼90 kDa and contained endoglycosidase H (EndoH)-sensitive glycans indicating localization in the early secretory pathway (Figure 1B). The interaction was not dependent on the formation of intermolecular disulfide bonds since the protein could be precipitated under reducing conditions (Figure 1A, lane 3). A similar result was obtained after pretreatment with the oxidant diamide (Figure 1A, lane 4). Upscaling of the co-immunoprecipitation (co-IP) experiment allowed protein identification using an EndoH-treated sample (Figure 1B). After excision of the protein band from a sodium dodecyl sulfate-polyacrylamide gel electrophoresis (SDS-PAGE) gel and trypsin digestion, mass spectrometry identified two proteins: SEL1L (as previously published [15]) and a hitherto uncharacterized protein (RefSeq: NP_065835; gene name: KIAA1344) (Figure S1). Based on its mobility by SDS-PAGE and its ER localization (see below), we named this protein ERp90. Since we identified both SEL1L and ERp90 from the same band of the EndoH-treated sample, a more detailed analysis necessitated the use of an ERp90 antibody. Thus, we raised an antibody against a single purified domain of ERp90 (Trx3; see below) expressed in *E. coli*. After affinity purification, this antiserum recognized the antigen (data not shown), but did not detect a band corresponding to endogenous ERp90 by Western blot analysis of cell lysates. However, we were able to re-precipitate endogenous ERp90 from lysates of radioactively labeled A11 cells following IP of ERFAD-HA (Fig. 1C, lane 4). Using this approach, and comparing to the migration of SEL1L, we could demonstrate that the glycosylated 90 kDa band coprecipitating with ERFAD-HA was ERp90 (Fig. 1C).

### ERp90 is a member of the PDI family

**Figure 2:**
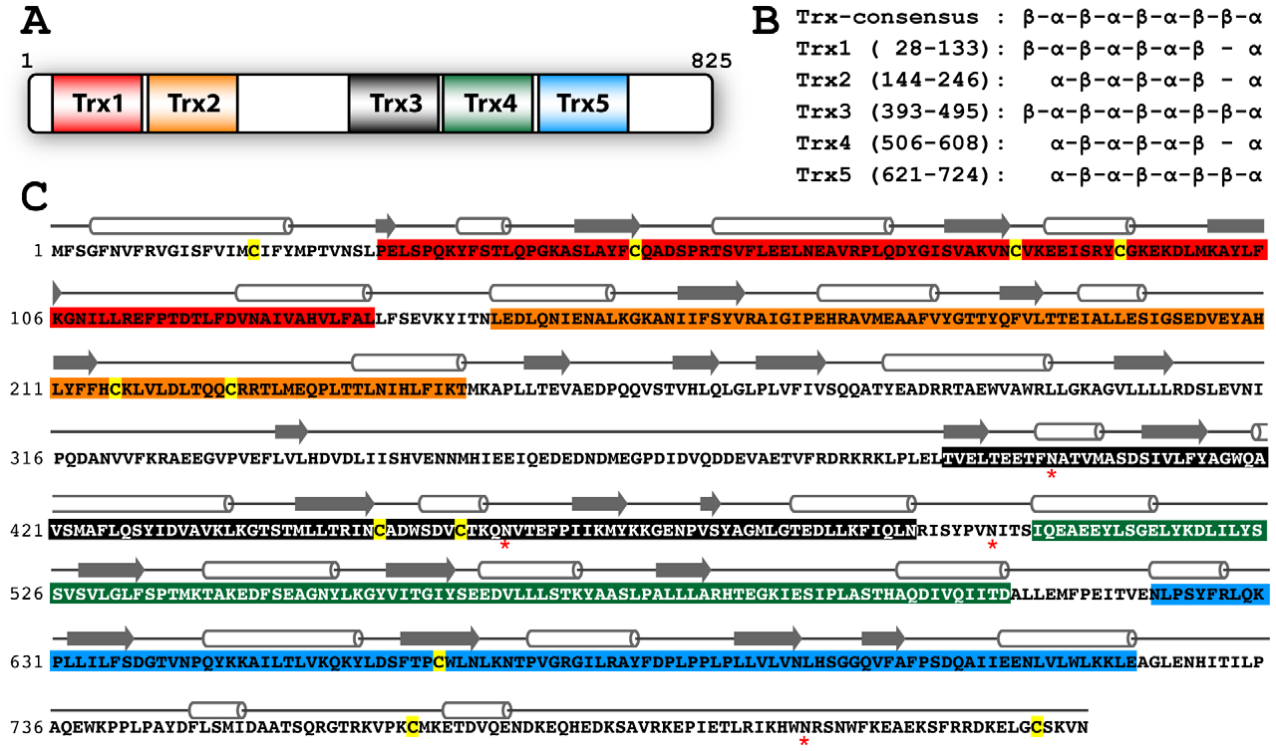
ERp90 contains five predicted Trx domains. (A) Domain organization of the human ERp90 protein based on sequence analysis. The positions of the five predicted Trx-like domains (Trx1-5) are depicted. (B) Secondary structure predictions for Trx1-5 in comparison to a Trx consensus domain. The secondary structure elements were predicted with PSIPRED [26], APSSP2 [27], and PROFseq [28]. The resulting sequence borders of the individual domains are given in the parentheses. (C) The amino acid sequence of ERp90 with the five Trx domains color-coded as in (A). Above the sequence, the location of predicted secondary structure elements are shown (cylinders indicate α-helices, and arrows β-strands). The N-terminal helix is part of the signal sequence. Cysteines are colored yellow, and the asterisks (*) mark four consensus sites for N-glycosylation.

The human ERp90 cDNA encodes a protein of 825 amino acids (Figure 2A and 2C). The N-terminal 27 residues are predicted to constitute an ER signal sequence, whereas no obvious ER retrieval motif is present at the C-terminus (see below). The mature ERp90 protein has a calculated molecular mass of 90.5 kDa. By secondary structure prediction we identified five potential Trx-like domains (Trx1-5) in ERp90 (Figure 2B). Such domains are a hallmark of proteins of the PDI family [11]. However, none of the five Trx domains in ERp90 contain the typical active-site CXXC motif of redox-active Trx-like domains. BLAST searching revealed the PDI-family member ERp57 as the closest homologue of ERp90. Specifically, similarity is observed between the Trx3-5 domains of ERp90 and the **abb'** domains of ERp57, with the ERp90 Trx3 and ERp57**a** domains showing the highest sequence similarity (Figure S2). It is noteworthy that the regular secondary structure elements determined experimentally in ERp57**abb'** show a high degree of conservation with those predicted in the corresponding region of ERp90 (Figure S2), supporting the assignment of the ERp90 Trx3-5 domains.

Database searching revealed orthologs of ERp90 in a number of vertebrates, urochordates and in *S. purpuratus* (a sea urchin), but not in the model organisms *S. cerevisiae*, *D. melanogaster* and *C. elegans*. An alignment of different ERp90 orthologs revealed high conservation over almost the complete ERp90 sequence (Figure S3). For instance, the sequence identity between the human (residues 42-819) and *Xenopus tropicalis* sequences was 48%.

Human ERp90 contains a total of 10 cysteine residues in the mature protein and four predicted N-glycosylation sites (Figure 2C). Three of the five predicted Trx-like domains contain short conserved di-cysteine motifs (Trx1: CX_8_C, Trx2: CX_9_C, Trx3: CX_6_C; Figure 2C and S3). In Trx1 and Trx3 these motifs are located in equivalent positions with the first cysteine placed in the loop following β_3_ and the second positioned in α_3_. Several PDI proteins contain similar motifs in this position, e.g. ERp57**a** (Figure S2) where the two cysteines form a structural disulfide. The CX_9_C motif in Trx2 localizes in a different position to a predicted loop region further C-terminal in the sequence (see Fig. 2C). In addition, a seventh cysteine in position 664 (Trx5) is conserved in almost all orthologs (Figure S3). It is noteworthy that one of the four consensus sites for N-glycosylation in the human ERp90 sequence is also conserved in a high number of orthologs (N460 located in the predicted loop between α_3_ and β_4_ in Trx3), indicating an important structural and/or functional role.

### The human ERp90 protein contains a C-terminal extension compared to its orthologs

**Figure 3:**
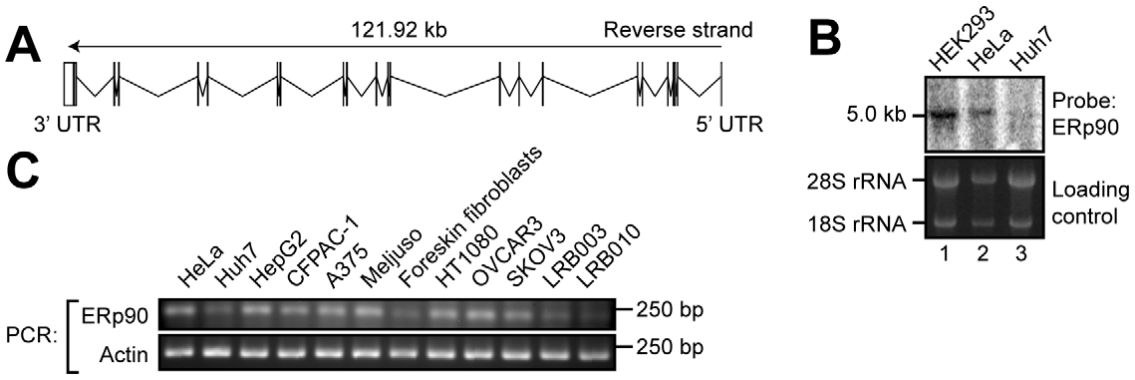
Transcriptional analysis of ERp90. (A) Exon-intron structure of the human ERp90 gene. Vertical lines indicate positions of the 21 predicted exons. (B) Northern blot detection of the ERp90 mRNA in human tissue-culture cells. A nitrocellulose membrane with RNA from three different human tissue-culture cell lines was hybridized with a radioactively labeled ERp90 cDNA probe (*top*). The rRNA signals served as loading controls (*bottom*). (C) RT-PCR analysis of ERp90. Total RNA was isolated from various human tissue-culture cells, reverse transcribed and amplified with primers specific for ERp90 and actin. HeLa: cervical epithelial carcinoma; Huh7, HepG2: hepatocellular carcinoma; CF-PAC-1: pancreatic adeno carcinoma; A375, Meljuso: melanoma; HT1080: fibrosarcoma breast cancer; OVCAR3, SKOV3: ovarian epithelial carcinoma; LRB003, LRB010: embryonic stem cells.

The ERp90 gene is located on chromosome 14q22.1, and is predicted to cover 121.91 kb of genomic DNA and contain 21 exons. The transcript length is predicted to 4.56 kb excluding the polyA tail (Figure 3A). We confirmed this prediction by Northern blot analysis using probes directed against the ERp90 mRNA (Figure 3B). Here, we detected one clear band that migrated at approximately 5 kb. Subsequent *in silico* (not shown) and RT-PCR analyses showed a broad tissue and cell line distribution of human ERp90 transcripts (Figure 3C).

Curiously, the human ERp90 protein is predicted to contain an additional six amino acids at the C terminus compared to all other orthologs (Figure S3). Moreover, the C-terminal four residues of the orthologs display similarity to a canonical KDEL ER-retrieval sequence (Figure S3). To determine the sequence of the ERp90 C-terminus we first isolated total mRNA from both HEK293 and HT1080 cells, and performed reverse transcription. We then amplified a region of the ERp90 cDNA that covers the 3′ end of the ERp90 open reading frame and the beginning of the 3′ untranslated region. Subsequent sequencing of the PCR products revealed that the human ERp90 gene in both cell lines indeed does encode for a C-terminally extended form of the protein (data not shown).

### Identification of a disulfide bond in recombinant ERp90 Trx3

**Figure 4:**
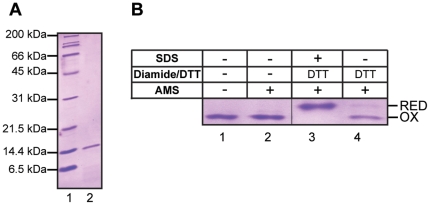
Purification and characterization of recombinant ERp90 Trx3. (A) Coomassie-stained gel of purified ERp90 Trx3. Lane 1, molecular weight marker; lane 2, purified ERp90 Trx3. (B) AMS shift assay performed on purified ERp90 Trx3. After treatment as detailed above the gel, the protein was TCA precipitated and resuspended in a denaturing buffer containing AMS (except for the control, lane 1). Note that DTT treatment was performed for 5 min at either 95°C (lane 3) or room temperature (lane 4). The samples were then subjected to SDS-PAGE and proteins were visualized by Coomassie-staining. The positions of oxidized (OX) and reduced (RED) ERp90 Trx3 in this assay are indicated. The hairline indicates where a lane has been removed from the gel.

For biochemical studies on ERp90 we first expressed the full-length protein in *E.coli*. However, we only succeeded in purifying small amounts of ERp90 extracted from inclusion bodies under denaturing conditions. We therefore decided to purify a single thioredoxin-like domain of ERp90. Due to the relatively high sequence similarity with ERp57**a** (Figure S2), we chose to work with ERp90 Trx3. Using this domain, we tested whether the cysteines in the CX_6_C motif form a disulfide bond. Here, we employed a gel shift assay based on alkylation with the maleimide reagent 4-acetamido-4′-maleimidylstilbene-2,2′-disulfonic acid (AMS) (Figure 4B). AMS efficiently reacts with free, but not with oxidized cysteines. In our experience, for proteins up to around 50 kDa modification of even just two cysteines with AMS (molecular weight: 499 Da) can add sufficient additional mass to introduce a detectable mobility shift by SDS-PAGE (see e.g. [17]). While the untreated ERp90 Trx3 domain did not shift upon AMS incubation, we observed full modification when ERp90 Trx3 was incubated with the reductant dithiothreitol (DTT) at 95°C under denaturing conditions prior to AMS treatment (Figure 4B, compare lanes 2 and 3). When incubating ERp90 Trx3 with DTT at 25°C under native conditions only a minor fraction of the Trx3 molecules became accessible for modification with AMS (Figure 4B, lane 4). These data indicate that the two cysteines of the CX_6_C motif in ERp90 Trx3 form a structural disulfide bond, as is the case for the cysteines of this motif in ERp57**a** [18]–[20], the **a**-domain of yeast PDI (Pdi1p**a**) [21], [22] and in all six Trx-like domains of ERdj5 [23].

### ERp90 is a soluble glycoprotein of the ER

**Figure 5:**
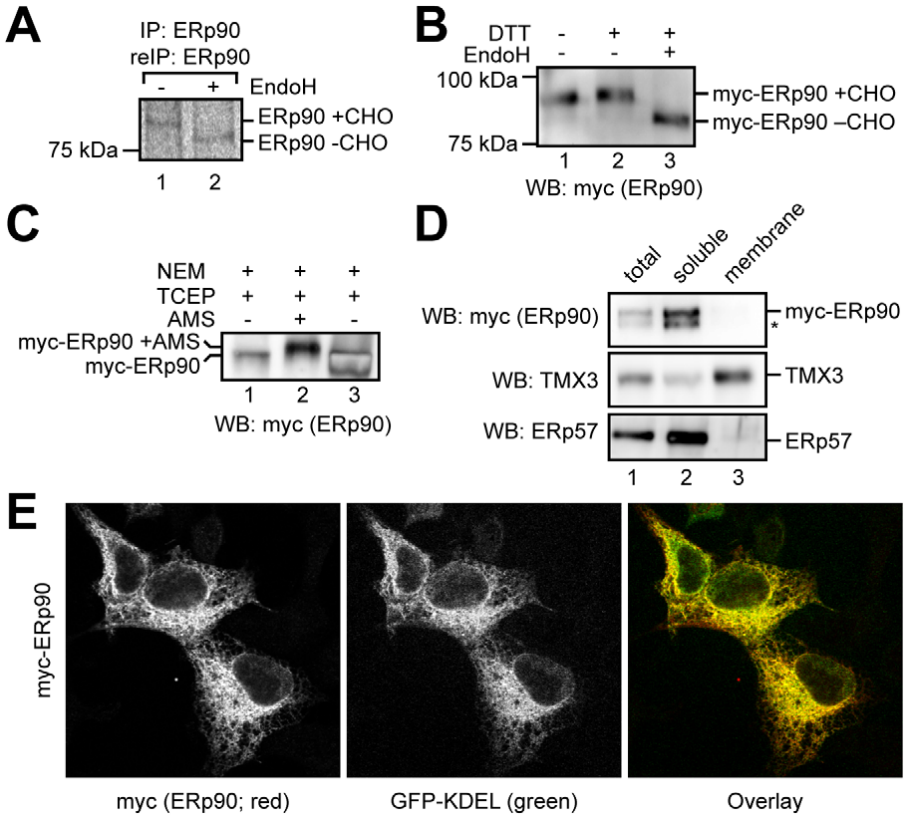
ERp90 is a soluble ER-glycoprotein. (A) Glycosylation state of ERp90. Lysates of pulse-labeled HEK293 cells were subjected to IP and re-IP with anti-ERp90. CHO, N-glycans. (B) Glycosylation and oxidation state of myc-ERp90. HEK293 cells transiently overexpressing myc-ERp90 were lysed after preincubation with the cell-permeable alkylating agent NEM to block free cysteines. The lysate was treated as indicated and extracts were analyzed by anti-myc Western blotting. CHO, N-glycans. (C) Oxidation state of myc-ERp90. HEK293 cells transiently overexpressing myc-ERp90 were lysed after preincubation with the cell-permeable alkylating agent NEM to block free cysteines. The lysate was treated with the reductant TCEP. Then, originally oxidized cysteines were modified with AMS. The resulting samples were analyzed by Western blotting with anti-myc. (D) Subcellular fractionation of HEK293 cells transiently expressing myc-ERp90. Crude membranes were isolated and extracted with sodium carbonate. The soluble and insoluble fractions were separated by ultracentrifugation through a sucrose cushion. The distribution of myc-ERp90, ERp57 (a soluble ER protein) and TMX3 (an ER membrane protein) was visualized by Western blotting. Asterisk, background band. (E) Confocal immunofluorescence microscopy of HEK293 cells transiently expressing myc-ERp90. HEK293 cells transiently co-expressing myc-ERp90 (red) and GFP-KDEL (green) were fixed, and stained with anti-myc. A merged image is shown in the right panel.

To analyze endogenous ERp90 in cells, we used our ERp90 antiserum in a double IP approach (see Material and Methods). Thereby, we were able to precipitate endogenous ERp90 as well as overexpressed myc-ERp90 from lysates of radioactively labeled cells (Figure 5A and S4, respectively). As expected for an ER protein with four potential N-glycosylation sites, the band representing endogenous ERp90 showed increased mobility upon EndoH cleavage (Figure 5A). Due to the rather low quality of the ERp90 antiserum we used transient expression of myc-tagged ERp90 in HEK293 cells for further experiments. The myc-ERp90 used was the full-length human protein including the C-terminal extension that we verified by RT-PCR (see above and Figure S3).

In accordance with the results for endogenous ERp90 we found that myc-ERp90 migrated at ∼95 kDa (Figure 5B). Myc-ERp90 displayed EndoH sensitivity and, upon DTT treatment, decreased gel mobility was observed (Figure 5B). Thus, at least some of the 10 cysteines in mature ERp90 were engaged in intramolecular disulfide bonds. To confirm the presence of disulfide bonds in ERp90 we used an alternative approach that has previously been applied to visualize the CX_6_C disulfide bond in ERp57**a** [17]. We first blocked free cysteines *in situ* using the alkylating agent N-ethyl maleimide (NEM). After cell lysis, we then reduced disulfide-bonded cysteines and modified them with AMS. By this procedure, only cysteines that were oxidized in the cell become modified with AMS. This approach confirmed that some of the cysteines in ERp90 are engaged in disulfide bonds (Figure 5C), in agreement with the *in vitro* analysis of Trx3 (Fig. 4B) that was performed under comparable experimental conditions (i.e. SDS denaturation at high temperature, reduction and then modification with AMS).

Next, we used alkali extraction of crude membranes isolated from HEK293 cells expressing myc-ERp90 to investigate whether the protein was soluble or membrane-bound. The membranes were extracted using sodium carbonate at pH 11.3, and the soluble and insoluble fractions were separated by ultracentrifugation before analyzing the presence of myc-ERp90 in either fraction by Western blotting. We found all myc-ERp90 in the same fraction as ERp57 (the control for soluble proteins), but not in the fraction containing the membrane protein TMX3 (Figure 5D), clearly indicating ERp90 to be a soluble protein.

The EndoH sensitivity of ERp90 already indicated a localization of the protein to the secretory pathway. However, due to the absence of an ER-retrieval signal it remained unclear whether ERp90 indeed was an ER-resident protein. Using immunofluorescence microscopy we observed a reticular staining pattern for transiently expressed myc-ERp90 and co-localization with an ER-targeted variant of the green fluorescent protein (GFP-KDEL) (Figure 5E). At the same time, a small fraction of myc-ERp90 was detected in the culture medium (data not shown). However, we consider the latter finding to be a likely result of overexpression, which could well saturate the retention mechanism for ERp90. Overall, the data show that ERp90 is a soluble, disulfide-containing glycoprotein of the ER.

### ERp90 forms a stable interaction with ERFAD

**Figure 6:**
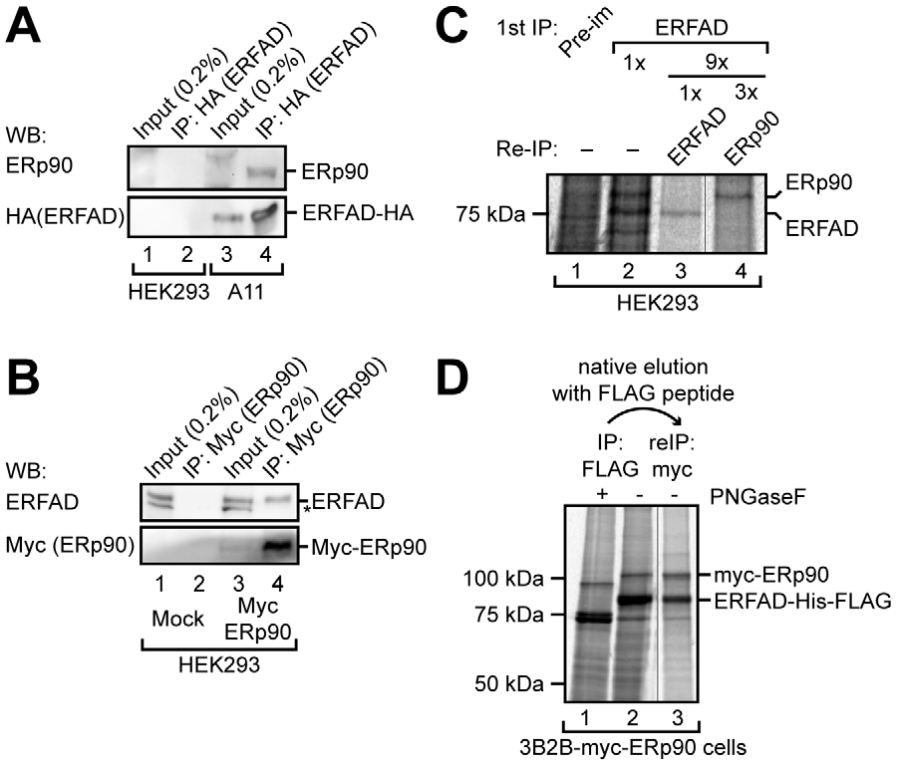
Analysis of the ERp90–ERFAD interaction. (A) ERp90 co-immunoprecipitates with ERFAD-HA. IPs from A11 or HEK293 cell lysates were performed with anti-HA, and analyzed by Western blotting with antibodies against ERp90 and the HA tag. 0.2% of the total lysate were directly analyzed as input. Asterisk, background band. (B) ERFAD co-immunoprecipitates with myc-ERp90. Anti-myc immunoprecipitates from lysates of HEK293 cells transiently overexpressing myc-ERp90 or an empty vector (mock) were subjected to Western blotting using the indicated antibodies. Asterisk, background band. (C) Endogenous ERFAD co-immunoprecipitates endogenous ERp90. ERFAD was immunoprecipitated from [^35^S] pulse-labeled HEK293 cells using a pre-immune serum or an ERFAD antiserum. The immunoprecipitate was either analyzed directly (lanes 1 and 2; one tenth of first immunoprecipitate loaded) or re-immunoprecipitated with antibodies against ERFAD (lane 3) or ERp90 (lane 4; three times the amount compared to the ERFAD re-immunoprecipitation in lane 3). The hairline indicates where a lane has been removed from the autoradiograph. (D) Overexpressed ERFAD-His-FLAG and myc-ERp90 can be co-purified. 3B2B-myc-ERp90 cells were [^35^S] pulse-labeled overnight and subjected to anti-FLAG IP. ERFAD-His-FLAG and its interacting partners were eluted with the FLAG peptide under native conditions (lanes 1 and 2), and subsequently re-immunoprecipitated with anti-myc under native conditions (lane 3).

Next, we wanted to characterize the interaction between ERFAD and ERp90 in more detail. We first immunoprecipitated ERFAD-HA from A11 cells using anti-HA, and then analyzed the precipitate by Western blotting with anti-ERp90 (Figure 6A). We also immunoprecipitated myc-tagged ERp90 overexpressed in HEK293 cells with anti-myc, and analyzed the eluted proteins by Western blotting with anti-ERFAD (Figure 6B). The results clearly showed that ERFAD-HA specifically co-precipitated endogenous ERp90 (Figure 6A, compare lanes 2 and 4) and myc-ERp90 co-precipitated endogenous ERFAD (Figure 6B, compare lanes 2 and 4). Importantly, IP of ERFAD from [^35^S]-methionine-labeled HEK293 cell lysates followed by re-IP against ERp90 confirmed the interaction between the two endogenous proteins (Figure 6C, lane 4). To obtain an indication of whether ERp90 and ERFAD interact directly, we tested if we could purify the complex of ERp90 and ERFAD from metabolically labeled cells that expressed tagged versions of both proteins (3B2B-myc-ERp90 cells expressing ERFAD-FLAG and myc-ERp90) (Figure 6D). First, we immunoprecipitated ERFAD-FLAG under native conditions taking care to elute the precipitated proteins under mild conditions using the FLAG peptide. We then performed a second native IP against the myc tag of ERp90. When we analyzed this latter precipitate we found that the predominant species purified were ERFAD-HA and myc-ERp90 (Figure 6D, lane 3) suggesting a stable and direct interaction between ERFAD and ERp90.

## Discussion

In this study we identify ERp90 as a new member of the PDI family and an interaction partner of the ERAD component ERFAD. Moreover, our preliminary data indicate that, like ERFAD [15], ERp90 can be crosslinked to a larger complex of proteins. At least certain of the bands in the two complexes immunoisolated with antibodies against ERFAD and ERp90, respectively, migrate at the same apparent molecular mass (data not shown). Based on these finding we cannot rule out entirely that ERFAD and ERp90 interact indirectly through other components of this complex. However, since the interaction between the two proteins withstands re-IP and because other proteins were much less abundant in the precipitate, we currently favor the idea that the interaction is direct.

The interaction between ERp90 and ERFAD strongly implies a functional link. This notion is reinforced by the presence of the two proteins in the same set of organisms. Since we did not succeed in performing siRNA-mediated knockdown experiments of ERp90 to study an involvement of ERp90 in ERAD, we can presently only speculate about the function of ERp90 in relation to ERFAD. While ERFAD likely catalyzes a redox reaction, ERp90 could recruit misfolded proteins to the complex by virtue of its redox-inactive thioredoxin-like domains. Substrates bound by the complex of ERFAD and ERp90 could then be delivered and handed over to SEL1L, a function resembling the one recently described for OS-9 and GRP94 [24]. Alternatively, ERFAD and ERp90 could work on substrates already bound by SEL1L or OS-9, and potentially also assist the transfer of substrates from either of these proteins to a retrotranslocation channel. Finally, ERp90 could perform a CXXC-independent redox function, conceivably in collaboration with the redox-active ERFAD, through the conserved C664 or even the CX_9_C motif in the Trx2. Future work should allow us to distinguish between these various possibilities.

## Materials and Methods

### Primers and plasmids

The ERp90 full-length cDNA clone fj00476s1 (KIAA1344) was obtained from the Kazusa DNA Research Institute, and the pCMV/myc/ER/GFP, pcDNA3, pcDNA5-FRT and pOG44 plasmids were from Invitrogen. The pFH255 plasmid was a gift from Dr. Lloyd Ruddock, University of Oulu. The following plasmids were constructed as described in Text S1: pcDNA5FRT/myc-ERp90, pcDNA3.hygro/myc-ERp90, and pMAL-c2X/MBP-Xa-ERp90Trx3.

### Antibodies

The following antibodies were used: anti-HA (16B12, Covance), anti-myc (9E10, Covance), anti-ERp57 (gift from A. Helenius, ETH Zurich, Switzerland), anti-TMX3 [25], anti-ERFAD [15] and anti-SEL1L (gift from H. Ploegh, Whitehead Institute, Cambridge, MA, USA). The secondary anti-rabbit and anti-mouse IgGs coupled to horseradish peroxidase were obtained from Pierce, and the Alexa Fluor 594 anti-mouse IgG from Invitrogen. A polyclonal serum against ERp90 (anti-ERp90) was generated by immunizing rabbits with the Trx3 domain of ERp90 purified from *E. coli* (see below). The obtained antiserum (56E) was affinity purified as follows: After SDS-PAGE, purified ERp90 Trx3 was blotted onto a nitrocellulose membrane. The membrane was stained with ponceau red and the region containing ERp90 Trx3 was cut out. The obtained nitrocellulose “strips” were blocked and then incubated with the anti-ERp90 serum overnight at 4°C. Strips were then washed, transferred to low salt buffer (10 mM Tris/HCl pH 7.5; 10 mM NaCl), and the antibodies eluted with a low pH buffer (100 mM glycine/HCl pH 2.8). The eluted antibody was immediately neutralized with 5 M NaOH and 1 M Tris, dialyzed overnight into PBS, concentrated, and supplemented with glycerol (to 50%).

### Cell lines

The generation of cell lines overexpressing ERFAD-HA (A11) and ERFAD-His-FLAG (3B2B) has been described [15]. The cell line stably expressing myc-ERp90 was generated using the Flp-In TREX system from Invitrogen (myc-ERp90 in HEK293-TREX, selected with 0.1 mg/ml hygromycin B and 15 µg/ml blasticidin). The 3B2B-myc-ERp90 cell line was generated by transfecting 3B2B cells with pcDNA3.hygro/myc-ERp90 and selecting with 0.1 mg/ml hygromycin B. All cells were cultured in modified Eagle medium alpha (Gibco) supplemented with 10% fetal calf serum (LabForce AG). Stable cell lines were cultured with the relevant antibiotics present.

### RT-PCR analysis, Western blotting, cell fractionation, EndoH digests, transient transfections and immunofluorescence

Total RNA was isolated (GenElute Total RNA kit, Sigma), and mRNA from 1 µg total RNA was reverse transcribed (Enhanced avian reverse transcriptase kit, Sigma). PCR reactions were performed with the equivalent of 200 ng total RNA using primers specific for ERp90 (for: cagaaattgcccttttggaa; rev: accagtggtaagcccagttg) and actin (for: ggacttcgagcaagagatgg; rev: agcactgtgttggcgtacag) and analyzed on 1% agarose gels. To investigate the identity of the 3′ region of the ERp90 mRNA, a PCR was performed using primers localized to the 3′ region of the last exon of the ERp90 mRNA and the 5′ region of the 3′ untranslated region of the ERp90 mRNA (for: cgaaagcttacctcc, rev: cgaaagctttttgcc). The PCR product was gel purified and sequenced. All other methods were performed as described previously [15].

### AMS modification of myc-ERp90 in HEK293-TREX cells

The experiment was performed as described in [17]. Cells were washed once with ice cold 20 mM NEM in PBS, incubated for 20 min in the same buffer and subsequently lysed in SDS-containing AMS modification buffer (0.05 M Tris-HCl, 2% SDS, 0.04% (w/v) bromocresol purple, pH 6.8). The cell lysate was then incubated at 95°C for 5–10 min and either analyzed directly or treated with TCEP at 25°C for 15 min. AMS was added to a final concentration of 20 mM, and incubated in the dark for 1 h at 25°C followed by the addition of Laemmli sample buffer. Samples were analyzed by SDS-PAGE and Western blot.

### *E. coli* expression, purification and AMS modification of recombinant ERp90 Trx3

A fusion protein of Maltose Binding Protein (MBP, positioned N-terminally) and the Trx3 domain of ERp90 (positioned C-terminally) connected with a Factor Xa (FXa) cleavage site was expressed in the SHuffle T7 Express strain of *E. coli* (New England Biolabs, MA, USA). SHuffle cells pre-transformed with a plasmid for Erv1 and DsbC expression regulated by an arabinose-inducible promotor (pFH255; kindly provided by Dr. Lloyd Ruddock, University of Oulu) were transformed with pMAL-c2X/MBP-Xa-ERp90 Trx3. LB broth containing 100 µg/ml ampicilin and 34 µg/ml chloramphenicol was inoculated with a single colony and grown overnight at 37°C. This culture was diluted to OD_600_ 0.05 and grown at 37°C. At OD_600_ ∼0.3, the incubation temperature was changed to 16°C. At OD_600_ ∼0.4, pre-expression of Erv1 and DsbC was induced by the addition of 0.5% w/v arabinose, and 40 min later, 0.5 mM IPTG was added to induce expression of ERp90 Trx3. Cells were grown overnight, pelleted, and resuspended in lysis buffer (20 mM Tris-HCl, 200 mM NaCl, 1 mM EDTA, 0.1% Triton X-100 (v/v), 100 µM phenylmethylsulfonyl fluoride, complete EDTA-free protease inhibitor tablet (Roche, Switzerland), pH 7.4). After sonication, the lysate was cleared by centrifugation at 14,500 *g* for 30 min at 4°C. The cleared lysate was loaded at RT onto an amylose resin (New England Biolabs, MA, USA) gravity-flow column pre-equilibrated with column buffer (20 mM Tris-HCl, 200 mM NaCl, pH 7.4). The column was extensively washed with column buffer and eluted in the same buffer containing 10 mM maltose. Pooled elution fractions were incubated in 10 mM NEM at 4°C for 1 h to block free thiols and dialyzed against FXa buffer (20 mM Tris-HCl, 50 mM NaCl, 1 mM CaCl_2_, pH 7.4). The MPB-ERp90 Trx3 fusion protein was cleaved with 3.5 U FXa (Qiagen, Germany) per mg protein for 12 h at RT. FXa was inactivated by the addition of 200 µM phenylmethylsulfonyl fluoride, and the cleaved protein was dialyzed against low salt buffer (20 mM Tris-HCl, 20 mM NaCl, pH 8.5) before loading it onto a 1 ml Mono Q HR 5/5 anion-exchange column (GE Healthcare, UK) mounted on an ÄKTAexplorer. Subsequent to washing in low salt buffer, proteins were eluted with a linear gradient developed over 40 min at 1 ml/min into high salt buffer (20 mM Tris-HCl, 0.5 M NaCl, pH 8.5). The correct molecular mass of ERp90 Trx3 was verified by matrix-assisted laser desorption/ionization mass spectrometry (expected: 13039 Da; experimentally determined: 13036 Da). The purified protein was used for gel shift assays after alkylation with AMS. Aliquots of ERp90 Trx3 in 20 mM Tris-HCl, 1 M NaCl, pH 8.5 were incubated under native or denaturing conditions (2% SDS), and treated with 10 mM DTT at 95°C (SDS-containing sample) or room temperature (RT) (all other samples) for 5 min. Thiol-disulfide exchange reactions were quenched and proteins were precipitated with 10% TCA followed by the addition of 0.1% deoxycholate. Samples were incubated on ice for 30 min, centrifuged 15 min at 4°C at 16,100 g and the supernatant was removed. The pellet was resolubilized and denatured in 0.5 M Tris-HCl, 2% SDS, 0.04% (w/v) bromocresol purple, pH 7.0 and incubated at 95°C for 2 min. Following the addition of 15 mM AMS, the samples were titrated with 1.5 M Tris-HCl, pH 8.8 until the color changed from yellow to purple (bromocresol purple changes color between pH 5.2–6.8), and incubated in the dark for 1 h at RT followed by the addition of Laemmli sample buffer. Samples were analyzed on Coomassie-stained 18% Tris-glycine SDS-PAGE gels.

### Metabolic labeling and immunoprecipitations

Pulse-chase experiments with [^35^S] Express protein labeling mix (PerkinElmer) and IP from cell lysates were performed as described [15]. The following lysis buffer was used for native IP: 50 mM HEPES/NaOH pH 7.2, 50 mM NaCl, 125 mM K-acetate, 2 mM MgCl_2_, 1 mM EDTA, 3% glycerol, 1% NP-40. To enrich endogenous ERp90 we performed sequential native and denaturing IPs. For the latter we used the following denaturing lysis buffer: 30 mM Tris/HCl pH 8.1, 100 mM NaCl, 5 mM EDTA, 1.6% SDS. Before adding antibodies, the buffer was diluted using 2.5% Triton X-100 to a final composition of 30 mM Tris/HCl pH 8.1, 100 mM NaCl, 5 mM EDTA, 0.4% SDS, 1.9% Triton X-100.

## Supporting Information

Figure S1 **Identification of ERp90 as an ERFAD-interacting protein.** (A) The protein sequence of full-length human ERp90. The peptides identified by mass spectrometry are indicated in bold lettering, and cover a total of 14% of the mature ERp90 protein sequence.
(EPS)

Figure S2 **Sequence alignment between ERp90Trx3-5 and ERp57 abb'.** Alignment of human ERp57 **abb'** domains with the three thioredoxin-like domains (Trx3-5) closest to the C-terminus of human ERp90. The alignment was performed with Muscle (1). The secondary structure of ERp90 was predicted as described in the legend for Figure 2. The secondary structure of ERp57 **abb'** domains is based on the structure solved by Dong and colleagues (2). The domains of ERp57 and ERp90 are indicated with colored lines and cysteine residues are highlighted in yellow.
(EPS)

Figure S3 **Evolutionary conservation of ERp90.** A multiple sequence alignment of the ERp90 protein was performed with Muscle (1) using the following database entries: *Homo sapiens* (accession number Q9P2K2.4), *Pan troglodytes* (accession number XP_001158742.1), *Macaca mulatta* (accession number XP_001103706.1), *Canis familiaris* (accession number XP_537446.2), *Bos taurus* (accession number XP_616195.4), *Mus musculus* (accession number BAD32431.1), *Rattus norvegicus* (accession number XP_001072487.1), *Gallus gallus* (accession number XP_421472.2), *Taeniopygia guttata* (accession number XP_002200455.1), *Xenopus tropicalis* (accession number NP_001072460.1), *Danio rerio* (accession number XP_685017.2), *Branchiostoma floridae* (accession number XP_002612374.1), *Saccoglossus kowalevskii* (accession number XP_002741184.1), *Strongylocentrotus purpuratus* (residue number 468–1288; accession number XP_001190577.1), and *Ciona intestinalis* (Ensembl Peptide ID ENSCINP00000018039). Black boxes indicate amino acid identities, and gray boxes show amino acid similarities when found in at least 8 of the 15 sequences. Cysteine residues are shown in yellow. The predicted signal sequence, N-glycosylation sites (*) and the thioredoxin-like domains of human ERp90 are depicted.
(PDF)

Figure S4 **Specificity of the antibody against ERp90.** Test of anti-ERp90 on cell lines overexpressing myc-ERp90. Cells stably expressing myc-ERp90 were [^35^S] pulse-labeled for 16 hours, Triton X-100 lysates immunoprecipitated either with anti-myc (lanes 1 and 2) or by the IP-reIP approach with anti-ERp90 (lane 3), and samples separated by reducing SDS-PAGE. Part of the eluate was deglycosylated by treatment with PNGaseF. The position of myc-ERp90 is indicated. CHO, N-glycans.
(EPS)

Text S1 **An overview of the cloning work performed to construct the pcDNA5FRT/myc-ERp90, pcDNA3.hygro/myc-ERp90, and pMAL-c2X/MBP-Xa-ERp90Trx3 plasmids.**
(DOC)
