# Supplementary material for: Identification of the PDI-Family Member ERp90 as an Interaction Partner of ERFAD
Source: PLoS One. 2011 Feb 16;6(2):e17037. doi: 10.1371/journal.pone.0017037 (PMC3040216; doi:10.1371/journal.pone.0017037)
Supplement: Figure S3 — Evolutionary conservation of ERp90. A multiple sequence alignment of the ERp90 protein was performed with Muscle (1) using the following database entries: Homo sapiens (accession number Q9P2K2.4), Pan troglodytes (accession number XP_001158742.1), Macaca mulatta (accession number XP_001103706.1), Canis familiaris (accession number XP_537446.2), Bos taurus (accession number XP_616195.4), Mus musculus (accession number BAD32431.1), Rattus norvegicus (accession number XP_001072487.1), Gallus gallus (accession number XP_421472.2), Taeniopygia guttata (accession number XP_002200455.1), Xenopus tropicalis (accession number NP_001072460.1), Danio rerio (accession number XP_685017.2), Branchiostoma floridae (accession number XP_002612374.1), Saccoglossus kowalevskii (accession number XP_002741184.1), Strongylocentrotus purpuratus (residue number 468–1288; accession number XP_001190577.1), and Ciona intestinalis (Ensembl Peptide ID ENSCINP00000018039). Black boxes indicate amino acid identities, and gray boxes show amino acid similarities when found in at least 8 of the 15 sequences. Cysteine residues are shown in yellow. The predicted signal sequence, N-glycosylation sites (*) and the thioredoxin-like domains of human ERp90 are depicted. (PDF) [file pone.0017037.s003.pdf]

Signal peptide

```
H.s.      1 -----------------------------------------------MFSGFNVFRVGISFVIMCIFYMPTVNSLPELSP
P.t.      1 -----------------------------------------------MFSGFNVFRVGISFVIMCIFYMPTVNSLPELSP
M.mul.    1 -----------------------------------------MQLQLMMSSGFGVFRVGISFVIMCSFYMPTVDSLLELSP
C.f.      1 ----------------------------------------------MMSSSFNIFRVGISFVIMCIFYKSAVDSLSELSP
B.t.      1 ---------------------------------------------------------------MCIFYTSAVDS-PELSP
M.mus.    1 ---------------------------NTAVKKTDKSPGTRLQAQLMMSSGFSVFRAGVALVLMCSFYKSTEDSLPELTP
R.n.      1 ------------MNQSTSGSSITSGVRTQQVKKTDKSPGTRIQPQLTMSSGLRVFRAGVALVLMCNFYTSTEDSLPELSP
G.g.      1 --------------------------------------------------------MTGAVRGSPQCLGPAALRVPAGAG
T.g.      1 ----------------------------------------------------------------------------MGTP
X.t.      1 --------------------------------------------------------------------------------
D.r.      1 -----------------------------------------------------MCTVYLVTFVFILTAEQCFSKLMEDSG
B.f.      1 -------------------------------------------MASYCKAFLLICLVALLATCTLVVSKELGVVNTVPSA
S.k.      1 ---------------------------------------------MAALRKCSWAVLVLTNLAVFATATDENLLFNPLKS
S.p.    468 --------------------------------------------------------------------------EAENVA
C.i.      1 ----------------------------------------------MSVKMLIIFCLCIATANAANQHDDFTFPQLSDND
```

Trx1

```
H.s.     34 QKYFSTLQPGKASLAYFCQAD--SPRTSVFLEELNEAVRPLQDYGISVAKVN---------CVKEEISRYCGKEKDLM-KA
P.t.     34 QKYFSTLQPGKASLAYFCQAD--SPRTSVFLEELNEAVRPLQDYGISVAKVN---------CVKEEISRYCGKEKDLM-KA
M.mul.   40 QKYFSTLQPGKASLAYFCQAD--SPRTSVFLEELNEAVRPLQDYGISVAKVN---------CVKEEISRYCGKEKELM-KA
C.f.     35 QKYFSTLQPGKASLAYFCQAD--SPRTSIFLEELNEAVTPLQDYGISVAKVN---------CVKEETSRYCGKEKDLM-KA
B.t.     17 QTFFSTLQPGKATLVYFCQAE--SLRTSMFLEELNEAAKPLQDYGISVAKVN---------CVTEETSRYCGSEKDLI-KA
M.mus.   54 QQYFSTLQPGKASLVYFCQVG--SLSNSVFLEELKEAVKPLQDYGISVAKVT---------CVEEEASRYCGEEEGLM-KA
R.n.     69 QQYFSSLQPGKASLAYFCQDG--SLSNSLFLEELKEAIRPLQDYGISVAKVT---------CVKEEASRYCGEEESLM-KA
G.g.     25 RGIWHQIPVAIDTAQKPQQAV--SPGAQPFLEQIENSVEALHDYGISVVKVNENKEWDRVKCPKEDVSRYCGKENALR-KA
T.g.      5 GCPSWIGTIRVPHGSGTSRLQ--DGDARPFWEQIESSAEALQDYGVSVVKVN---------CPKEDVSRYCGKENALR-QA
X.t.      1 -------MPGKTSLLYFTNPA--FPSNIIFLDELQKSAGALQDYGISVAKVN---------CMKEDILKYCGEE-----NA
D.r.     28 QNLMERMQTGKTSFIYFGNHV--NPTIELFIEQLQMSADALEDYGIAVVMVN---------CSKDDIAKYCTGEKLLT-KA
B.f.     38 EFEDFIGSPSTLTAVYFYKRR--LQRLKHFLESFDAAASFMKLYQVQFGRVN---------CDIDPVAEYCDKPDVDM-SV
S.k.     36 KDLNDFVSEPFVRVVYFFKGELPLQQPDRFLLNFKQASDILEDYGVKTATLH---------CKNEAMNEYCSRKHSVAESV
S.p.    474 GVLEAILGMIRDPHYRVIGGS--VAHSKTFQGHFSEAGDYLKDFGVFLGTVN---------CISDPVSEYCLKADASL-NL
C.i.     35 VSLHKQSNSGFGLLLMHNKDQ--TEDFSAALHSILDASESLNKNGAIVGVLN---------CN-ESTDVLCEEKEE---KL
```

```
H.s.    103 YLFKGNILLREFPTDTLFDVNAIVAHVLFALLFSEVKYITN-LEDLQNIENALKGKANIIFSYVRAIGIPEHRAVMEAAF
P.t.    103 YLFKGNILLREFPTDTLFDVNAIVAHVLFALLFNEVKYITN-LEDLQNIENALKGKANIIFSYVRAIGIPEHRAVMEAAF
M.mul.  109 YLFKGNILLREFPTDTLFDVNAIVAHVLFALLFNEVKYITN-LEDLQNIENALKGKANIIFSYVRATGIPEHRAVMEAAF
C.f.    104 YLFRGNILLREFPTDVLFDVNAIVAHVLFALLFNEVKYITT-LEDLQNIENALKGKANIAFAYVRAIGTPEHRAVMEAAF
B.t.     86 YLFRGNIPLREFPTDTLFDVDAIVAHVLFALLFNEVKYITT-LGDLQNIENALKGKRNIAFSYVRAIGTPEHRAVMEAAF
M.mus.  123 YLFRGNILLREFPTDILFDVNAIIAHVLFALLFNEVKYITT-LEDLHSIENSLKGKSNMIFSYVEAIGTPEHRAVMEAAF
R.n.    138 YLFRGSILLREFPTDILFDVNAIIAHVLFALLFNEVKYITT-LEDLHSIENSLKGKSNMIFSYVEAIGTPEHRAVMEAAF
G.g.    103 YLFRGNVLIREFPTDALFDVNSIVANVLFALLFHEVKYVET-LTDLQNIENALKGKSNMVFAYVPATGTAEHRAVMEVAF
T.g.     74 FLFRGNRLIREFPTDALFDVNSIVANVLFALLFNEVKYVET-LADLENIENLLKGKSNMVFAYVPATGTAEHRAVMEAAF
X.t.     59 YLFRGAKLVREFPTDALFDVDSIVANVLFVLLYNEVKYITT-SMEHQNIESAMKGKKDLIFAYVQAIGIPEHRSVMETAF
D.r.     97 YLFRGSEVLRSFDIDTVFDVNAIVSHVLFSVLFNEVLYVHT-PAELLGVESAAKQNSDIVVGHIPVLGLPEHRALMETAF
B.f.    107 FAFRNDRVEAKFALDTMYDRDAIASNIFQVLLSSEVPVLQS-WIDLSHQQQRYMGDKDIIFSWQKAIGTYQHRVFMEFAF
S.k.    108 FIFRGGNLLLEIQLASMFNIDAIVSNILQMVMLGEVPIVSD-EEKLQQIMDMNKGKQDVLYSYQRAVGTLEHRVFMEVAF
S.p.    543 YIFRHGHFFITLAYDTLFSVDSIVSNILHLVLLIDVPIIAG-PSDLLEKQDRYKGKKDIIVGYVPAVGITADRALMEVAF
C.i.    101 VVYKGGEFITSLDLGHLSTPETAEGNILTQLYDSTFPDISEVEVFNKVLEEAKSNKKNLLLAVT-EPATPLHQYLLKTAL
```

Trx2

```
H.s.    182 VYGTTYQFVLTTEIALLESIGSE-----DVEYAHLYFFHCKLVLDL---TQQCRRTLMEQPLTTLNIHLFIKTMKAPLLT
P.t.    182 VYGTTYQFVLTTEIALLESIGSE-----DVEYAHLYFFHCKLVLDL---TQQCRRTLMEQPLTTLNIHLFIKTMKAPLLT
M.mul.  188 VYGTTYQFVLTTEIALLESIGSE-----DVEYAHLYFFHCKLVLDL---TQQCRRTLMEQPLTTLNIHLFIKTMKAPLLT
C.f.    183 VYGTTYQFVLTTETALLESIGNE-----DIENAHLYFFHCKQVLDL---TLQCRRTLMEQPLTTLNIHQFIKTMEAPLLT
B.t.    165 VYGTTYQFVLTTEIAVLESIGPE-----DIEYPHLYFFYCKQVLDL---TQPCRRILMKQSLTTLNIHRFIKTMEAPLLT
M.mus.  202 VYGTSYQFALTTEIALLENIGSE-----SIEHAHLYFFHCKLVLDL---TEHCRRTLMEQPLTTLNIHVFVKTMNAPLLM
R.n.    217 VYGTSYQFALTTEIALLEDVGSE-----NIEHAHLYFFHCKVVSDV---TEHCRRTLMGPPLAAVEVHKFVKTMEAPLLT
G.g.    182 VYGTVHQFVLTTEATLLKDAGSND-P--DVSSAQLLFCHCQQATDL---AQPCRRTPLEHDLTVLNIHRHLKLMGAPLVT
T.g.    153 VYGTIHQFVLTTEAALLKATSHDD-P--DVSSAWLSFCHCQRATEA---AQPCRRTALAQPLTLLSIHRHLKLMGAPLVT
X.t.    138 VYGSKYQFILTTETKLLENIS-A-----SLDSSRLFFSHCKGIKTH---SQECPRTILEQPITTVNIHRFLKLMDAPLVM
D.r.    176 VYGTKYQFVQTTGSLVLKRMGVVN-P--SSSQARLWFLHCKSVSLQ---SDPCPSTAMSKPINTINIHTFLQLMEAPLVT
B.f.    186 AYGSTYKFAFATNKKIAEDADLRDAK--SVKEPAIWVLHCSEEEKT---AKTCRQTRFQHSIVLADVLAFVRTLKTPYYV
S.k.    187 VYSNQYAFALSTSTEVARLISST-----DVRHPTIWYLQCKSVSPMDASTKSCPVLRYTGPINLETFSKYMKLISMPNVL
S.p.    622 AYRHKYVFLVTTNRNLMEEEQEKKPSLNRLPDPLVAISQCSKA-QP---HQDCVWAKYNGPVTLESLSIMMKVLELPAMV
C.i.    180 STDADLMFAYTQNSDLSFFLGDTGRV--MNKNGELWVVMCSV--ET---SGECPYFQYKGVMTAYGIKSLLAHMQLPYFS
```

```
                 β           β          β                 α                  β
H.s.     254 EVAEDPQQVSTVHLQLGL---PLVFIVS-QQATYEADRRTAEWVAWRLLGKAGVLLLLRDSLE-VNIPQDA---------
P.t.     254 EVAEDPQQVSTVHLQLGL---PLVFIVS-QQATYEADRRTAEWVAWRLLGKAGVLLLLRDSLE-VNIPQDA---------
M.mul.   260 EVAEDPQQVSTVHLQLGL---PLVFIVS-QQATYEADRRTAEWVAWRLLGKAGVLLLLRDSLE-VNIPQHA---------
C.f.     255 EVTEDPQQVSTIHLQLGL---PLVFIVS-QQATYEADRRTAEWVAWRLLGKAGVLLLLRDSLE-VDIPQHA---------
B.t.     237 EVAGDPTQVSTVHLQLGL---PLVFIVG-QQATYEADRTTAEWVAWRLLGKAGVLLLLRDSLE-VDIPHDT---------
M.mus.   274 EVAEDPQQVSTVHLQLGL---PLVFIIS-QRATQEADRRTAEWVAWHLLGKAGVLLLLRDSMD-VNIPQHA---------
R.n.     289 EVAEDPQLVSTIHLQLGL---PLVFIIS-QRATHEADRRTAEWVAWQLLGKAGVLLLMRDSAD-VNIPQHA---------
G.g.     256 EVAEDPEKVSTVHLQLGL---PLVFILS-QKETYEADRRTAEFLAWKLLGKAGVAILSRDVVD-LNILRRS---------
T.g.     227 EVAEDPEKISTVHLQLGL---PLVFILS-QKETYEADRRTAEFVAWQLLGKAGVALLSRDLVE-LNVLHRS---------
X.t.     209 DISGDPEKVTSVHLQLGL---PMVFILS-QQETYEFDRTTAEHVAWQLLGKAGIGILLREKAH-SSVPTNC---------
D.r.     250 EAVTDPSEVDVVHIHLSV---PVLYLFS-QPQTQHLDRDTAQTVALQLRGEVGVVLIHRDNPK-VKTPLKY---------
B.f.     261 DMPINQE--PTPYETMNL---HLVYVAT-DNSNEHEAREVADKLGRALRGQAGVILVNVDTASEITLESLA---------
S.k.     262 DFQESMLT-KSEFDDLQV---HRAYIFT-DAASYKSTQALTAELGSHFIGSMVFLIVNVNKLP-TYVVKQLGYGSQVTEV
S.p.     698 DMSEAGQ--SDVYKEHGI---STLYTIT-DPTNHEQALSLIVDVKDEYQGNLGFIVIDRDAVSD------A---------
C.i.     253 IQAENTNS-LSAHINSGLENGRDVAILTAGAGKLERAEKLSEMLCSVSYPYSK--CLFVDGQKV-ET---P---------

                              β
H.s.     320 -NVVFKRA--EEGVPVEFLVLHDV--------------------------------------
P.t.     320 -NVVFKRA--EEGVPVEFLVLHDV--------------------------------------
M.mul.   326 -NVVFKRA--EKGVPVEFLVLHDV--------------------------------------
C.f.     321 -NVVFRRA--EEGVPVEFLVLDDI--------------------------------------
B.t.     303 -NVIIRRA--EEDTQVEFLALSHI--------------------------------------
M.mus.   340 -NVAFRRA--EKDVPVEFLVLNDV--------------------------------------
R.n.     355 -NVAFRRA--EKDAPVEFQVLNDV--------------------------------------
G.g.     322 -NIALKTP--DEGVPIKYLVLEDT--------------------------------------
T.g.     293 -NVALKTP--DEGMPIQYLVLEDT--------------------------------------
X.t.     275 -NVAVKRP--NEDSPVQYMTMEET--------------------------------------
D.r.     316 -NAAYRLP--QED--VKYFTLSAP--------------------------------------
B.f.     326 -TDT-ER---LREVPVVAVQLYGQDTMHFMHHFSEGPLVEDL--------------------
S.k.     336 PSAAFRSY---GSNNLEYMSVKFTETNI----------------------------------
S.p.     757 -NLLADLK--GECIPACFVIQSHDQVISGEGIHDVSGLKAFIK-------------------
C.i.     317 -NVAIPNKIKDGRIYMSFKTQPDIEQEIVVGYLGSETLGEVEQHFQRLEQHRKILDRSLRFR

H.s.     341 -----------------DLIISHVENNM---HIE-EIQEDEDND-------MEGP--DIDVQDDEVAETVFRDRKRK-L-
P.t.     341 -----------------DLIISHVENNM---HIE-EIQEDEDND-------MEGP--DIDVQDDEVAETVFRDRKRK-L-
M.mul.   347 -----------------DSIISHVEHNT---HIE-EIQEDEDDD-------MEGP--DIDVQDDEVAETVFRDRKRK-L-
C.f.     342 -----------------DSLISHVESNV---HVE-EIQEDEDED-------TKSP--NMDVQDDEVAETVYRDRKRP-L-
B.t.     324 -----------------DLIVFHLESTM---NVE-EMVKYE--D-------MAEL--NIEIQDDEVEETVYRDRKRQ-L-
M.mus.   361 -----------------ELIISHVKNNM---HIE-EIQEDEGED-------MEGP--DLAVEDDEVAGTVYRDRKKP-L-
R.n.     376 -----------------ELIISHVKNNL---YFE-EIQEDEGED-------MEGP--DLAVEDDEVAGTVYRDRKRP-L-
G.g.     343 -----------------EEVITLVEDKS---KVK-QIQENEDEDEE-DEDEKEND--DQDVQDDQVVEAVSRDKKRE-L-
T.g.     314 -----------------DEVITLVEDKN---KVK-QIQEDEQEEEEEDEDEKENN--NQDIQDDQVVEAVTRDKKRE-L-
X.t.     296 -----------------QEIRNIIEKS----NME-QYNKSEETS--------VFA--TQEIQDDEVAEAVYRDRKRT-L-
D.r.     335 -----------------EEVVKLFKETL---LQK-DKTEDEEE------DDEHWS--DLDILDDEVSESVYRDRDLM-L-
B.f.     363 -----------------EVLLSEHRRST---ERK-KIIQDEDEG-------FDEP--DEEVQDDVVAEAVYRSRYAQ-L-
S.k.     361 -----------------RNFLKHVLDTF---NEE-TMDERLYTD---NHPIFEVP--PQEIQDDPVAEDAYLIRNKK-F-
S.p.     697 --------------INIQRVFSYLDDPNQGDEND-VMGEDGDDD-------GDVP--VQERQDDLVQQATVHSRLVI-E-
C.i.     378 YPDGDLPPSKRGPIRGSEEVD-------GDDENEVLVAADEDSY-------NEDPKYAQEVQDDAVMHAVHTNKELKDLY

                        Trx3
                 β       * α        β              α                                 β
H.s.     389 --PLEL-TVELTEETFNATVMA-SDSIVLFYAGWQAVSMAFLQSYIDVAVKLK-----GTST-----------MLLTRIN
P.t.     389 --PLEL-TVELTEETFNATVMA-SDSIVLFYAGWQAVSMAFLQSYIDVAVKLK-----GTST-----------MLLTRIN
M.mul.   395 --PLEL-TVELTEETFNATVTA-SDSMVLFYAGWQAVSMAFLQSYVDVAVKLK-----GTST-----------MLLTRIN
C.f.     390 --PLEL-TVELTEETFNTTVVA-SDSIVLFYAGWQAVSMAFLQSYIDLAIKLK-----GTST-----------MLLTRVN
B.t.     370 --PLEL-TLELTEETFHATVTA-SDSIVLFYAGWQAVSMAFLQSYIDVAIKLK-----GTST-----------MLLARVN
M.mus.   409 --PLEL-SVELTEETFNTTVMT-SDSIVLFYATWHAVSMAFLQSYIDVAIKLK-----GRST-----------ILLTRIN
R.n.     424 --PLGL-LVELTEETFNTTVMA-SDTLVLFYATWHAVSMAFLQSYTDVAVKLK-----GRST-----------ILLTRIN
G.g.     397 --PLDQ-ILTLTEETFHSAMLEAAQTVVLFYASWEAVSLVVLQSYTEVADHLK-----GTQG-----------ILLSRVN
T.g.     369 --PLEQ-ILVLTEENFHSSLSEASQTVVLFYASWEAVSLAVLRSYSEVAAHLK-----GAPG-----------VLLSRVN
X.t.     342 --PLHL-VPSLTDENFKHVLTNPPMSMILYYASWEAVSLTLLQTFVHMAEKYK-----DTLD-----------MILARVN
D.r.     384 --DLEP-VTELTADTFQTAIKQNEITVVLFYFKWDAVCMAFIQSYVEVAEAVE-----GKSTSLNNIAEYVNGVELAAVD
B.f.     411 --DLTY-APALTDKTFPAMLKEKSLLVVVFYLSWEPRSAAFLASYSEASLALRD---ETSNS-----------SPLARVA
S.k.     413 --NTTS-IPALTDKTFPAMLSEKNLFLVMFYVQWDPRTQVFMEHYAEAGRMLEHM--TSDNT-----------SPLARVN
S.p.     851 --PKKYIISSLTDKTFPEFTSQSHLTSVLFTMEWNPRSLAFLDSFDSAAESILAFFSSESQE-----------PPLARVE
C.i.     444 LYDFKS-YSRLTDKTYETVTNGNEYAVILFTHEFNPKALAALSSFIGIHRTYK-------EG-----------SPLHRVE
```

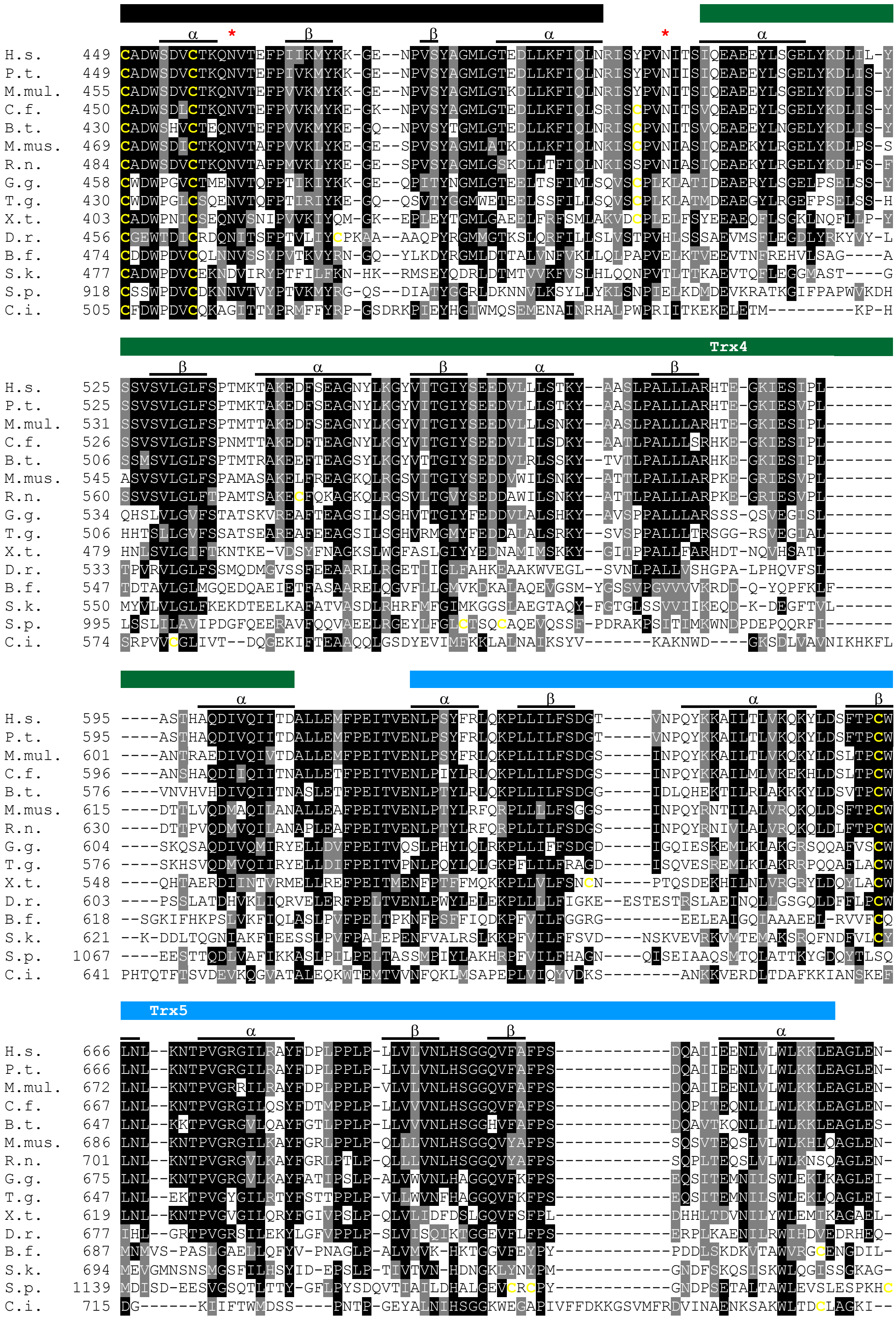

α * β β α * α
H.s. 449 CADWSDVCTKQNVTEFPIIKMYKK-GE--NPVSYAGMLGTEDLLKFIQLNRISYPVNITSIQEAEEYLSGELYKDLIL-Y
P.t. 449 CADWSDVCTKQNVTEFPIVKMYKK-GE--NPVSYAGMLGTEDLLKFIQLNRISYPVNIISIQEAEEYLSGELYKDLIS-Y
M.mul. 455 CADWSDVCTKQNVTEFPVVKMYKK-GE--NPVSYAGMLGTKDLLKFIQLNRISYPVNITSIQEAEEYLSGELYKDLIS-Y
C.f. 450 CADWSDICTKQNVTEFPVVKMYKE-GK--NPVSYAGMLGTEDLLKFIQLSRISYPVNITSVQEAEEYLSGELYKDLIS-Y
B.t. 430 CADWSHVCTEQNVTEFPVVKMYKE-GQ--NPVSYTGMLGTEDLLKFIQLNRISCPVNITSVQEAEEYLNGELYKDLIS-Y
M.mus. 469 CADWSDICTKQNVTAFPVVKLYKE-GE--SPVSYAGMLATKDLLKFIQLNKISCPVNIASIQEAEKYLRGELYKDLPS-S
R.n. 484 CADWSDVCTKQNVTAFPMVKLYKE-GE--SPVSYAGMLGSKDLLTFIQLNKISSPVNIASIQEAEKYLRGELYKDLFS-F
G.g. 458 CWDWPGVCTMENVTQFPTIKIYKK-GE--QPITYNGMLGTEELTSFIMLSQVSCPLKLATIDEAERYLSGELPSELSS-Y
T.g. 430 CWDWPGICSQENVTQFPTIRIYKE-GQ--QSVTYGGMWETEELSSFILLSQVSCPLKLATMDEAEGYLRGEFPSELSS-H
X.t. 403 CADWPNICSEQNVSNIPVVKIYQM-GK--EPLEYTGMLGAEELFRFSMLAKVDCPLELFSYEEAEQFLSGKLNQFLLP-Y
D.r. 456 CGEWTDICRDQNITSFPTVLIYCPKAA--AAQPYRGMMGTKSLQRFILLSLVSTPVHLSSSAEVMSFLEGDLYRKYVY-L
B.f. 474 CDDWPDVCQLNNVSSYPVTKVYRN-GQ--YLKDYRGMLDTTALVNFVKLLQLPAPVELKTVEEVTNFREHVLSAG----A
S.k. 477 CADWPDVCEKNDVIRYPTFILFKN-HK--RMSEYQDRLDTMTVVKFVSLHLQQNPVTLTTKAEVTQFLEGGMAST----G
S.p. 918 CSSWPDVCDKNNVTVYPTVKMYRG-QS--DIATYGGRLDKNNVLKSYLLYKLSNPIELKDMDEVKRATKGIFPAPWVKDH
C.i. 505 CFDWPDVCQKAGITTYPRMFFYRP-GSDRKPIEYHGIWMQSEMENAINRHALPWPRIITKEKELETM---------KP-H
Trx4
β α β α β
H.s. 525 SSVSVLGLFSPTMKTAKEDFSEAGNYLKGYVITGIYSEEDVLLLSTKY--AASLPALLLARHTE-GKIESIPL-------
P.t. 525 SSVSVLGLFSPTMKTAKEDFSEAGNYLKGYVITGIYSEEDVLLLSTKY--AASLPALLLARHTE-GKIESVPL-------
M.mul. 531 SSVSVLGLFSPTMTTAKEDFSEAGNYLKGYVITGIYSEEDVLLLSNKY--AASLPALLLARHTE-GKIESIPL-------
C.f. 526 SSVSVLGLFSPNMTTAKEDFTEAGNYLKGYVITGIYSEEDVLILSDKY--AATLPALLLSRHKE-GKIESIPL-------
B.t. 506 SSMSVLGLFSPTMTRAKEEFTEAGSYLKGYVTTGIYSEEDVLRLSSKY--TVTLPALLLARHKE-GKIESIPL-------
M.mus. 545 ASVSVLGLFSPAMASAKELFREAGKQLRGSVITGIYSEDDVWILSNKY--ATTLPALLLARPKE-GRIESVPL-------
R.n. 560 SSVSVLGLFTPAMTSAKECFQKAGKQLRGSVLTGVYSEDDAWILSNKY--ATTLPALLLARPKE-GRIESVPL-------
G.g. 534 QHSLVLGVFSTATSKVREAFTEAGSILSGHVTTGIYFEDDVIALSHKY--AVSPPALLLARSSS-QSVEGISL-------
T.g. 506 HHTSLLGVFSSATSEAREAFEEAGSILSGHVRMGMYFEDDALALSRKY--SVSPPALLLTRSGG-RSVEGIAL-------
X.t. 479 HNLSVLGIFTKNTKE-VDSYFNAGKSLWGFASLGIYYEDNAMIMSKKY--GITPPALLFARHDT-NQVHSATL-------
D.r. 533 TPVRVLGLFSSMQDMGVSSFEEAARLLRGETIIGLFAHKEAAKWVEGL--SVNLPALLVSHGPA-LPHQVFSL-------
B.f. 547 TDTAVLGLMGQEDQAEIETFASAARELQGVFLLGMVKDKALAQEVGSM-YGSSVPGVVVVKRDD-Q-YQPFKLF------
S.k. 550 MYVLVLGLFKEKDTEELKAFATVASDLRHRFMFGIMKGGSLAEGTAQY-FGTGLSSVVIIKEQD-K-DEGFTVL------
S.p. 995 LSSLILAVIPDGFQEERAVFQQVAEELRGEYLFGLCTSQCAQEVQSSF-PDRAKPSITIMKWNDPDEPQQRFI-------
C.i. 574 SRPVVCGLIVT--DQGEKIFTEAAQQLGSDYEVIMFKKLALNAIKSYV-------KAKNWD----GKSDLVAVNIKHKFL
α α β α β
H.s. 595 ----ASTHAQDIVQIITDALLEMFPEITVENLPSYFRLQKPLLILFSDGT-----VNPQYKKAILTLVKQKYLDSFTPCW
P.t. 595 ----ASTHAQDIVQIITDALLEMFPEITVENLPSYFRLQKPLLILFSDGT-----VNPQYKKAILTLVKQKYLDSFTPCW
M.mul. 601 ----ANTRAEDIVQIVTDALLEMFPEITVENLPSYFRLQKPLLILFSDGS-----INPQYKKAILTLVKQKYLDSLTPCW
C.f. 596 ----ANSHAQDIIQIITNALLETFPEITVENLPIYLRLQKPLLILFSDGS-----INPQYKKAILMLVKEKHLDSLTPCW
B.t. 576 ----VNVHVHDIVQIITNASLETFPEITVENLPTYLRLQKPLLILFSDGG-----IDLQHEKTILRLAKKKYLDSVTPCW
M.mus. 615 ----DTTLVQDMAQILANALLEAFPEITVENLPTYLRFQRPLLLLFSGGS-----INPQYRNTILALVRQKQLDSFTPCW
R.n. 630 ----DTTPVQDMVQILANAPLEAFPEITVENLPTYLRFQRPLLILFSDGS-----INPQYRNIVLALVRQKQLDLFTPCW
G.g. 604 ----SKQSAQDIVQMIRYELLDVFPEITVQSLPHYLQLRKPLLIFFSDGD-----IGQIESKEMLKLAKGRSQQAFVSCW
T.g. 576 ----SKHSVQDMVQIIRYELLDIFPEITVPNLPQYLQLGKPFLILFRAGD-----ISQVESREMLKLAKRRPQQAFLACW
X.t. 548 ----QHTAERDIINTVRMELLREFPEITMENFPTFFMQKKPLLVLFSNCN-----PTQSDEKHILNLVRGRYLDQYLACW
D.r. 603 ----PSSLATDHVKLIQRVELERFPELTVENLPWYLELEKPLLLLFIGKE--ESTESTRSLAEINQLLGSGQLDFFLPCW
B.f. 618 --SGKIFHKPSIVKFIQLASLPVFPELTPKNFPSFFIQDKPFVILFGGRG--------EELEAIGQIAAAEEL-RVVFCQ
S.k. 621 --K-DDLTQGNIAKFIEESSLPVFPALEPENFVALRSLKKPFVILFFSVD----NSKVEVRKVMTEMAKSRQFNDFVLCY
S.p. 1067 ----EESTTQDIVAFIKKASLPILPELTASSMPIYLAKHRPFVILFHAGN----QISEIAAQSMTQLATTKYGDQYTLSQ
C.i. 641 PHTQTFTSVDEVKQGVATALEQKWTEMTVVNFQKLMSAPEPLVIQYVDKS--------ANKKVERDLTDAFKKIANSKEF
Trx5
α β β α
H.s. 666 LNL--KNTPVGRGILRAYFDPLPPLP-LLVLVNLHSGGQVFAFPS------------DQAIIEENLVLWLKKLEAGLEN-
P.t. 666 LNL--KNTPVGRGILRAYFDPLPPLP-LLVLVNLHSGGQVFAFPS------------DQAIIEENLVLWLKKLEAGLEN-
M.mul. 672 LNL--KNTPVGRRILRAYFDPLPPLP-VLVLVNLHSGGQVFAFPS------------DQAIIEENLVLWLKKLEAGLEN-
C.f. 667 LNL--KNTPVGRGILQSYFDTMPPLP-LLVVVNLHSGGQVFAFPS------------DQPITEQNLILWLKKLEAGLEN-
B.t. 647 LNL--KKTPVGRGVLQAYFGTLPPLP-LLVVVNLHSGGHVFAFPS------------DQAVTKQNLILWLKKLEAGLES-
M.mus. 686 LNL--KNTPVGRGILKAYFGRLPPLP-QLLLVNLHSGGQVYAFPS------------SQSVTEQSLVLWLKHLQAGLEN-
R.n. 701 LNL--KNTPVGRGVLKAYFGRLPTLP-QLLLVNLHSGGQVYAFPS------------SQPLTEQSLVLWLKNSQAGLEN-
G.g. 675 LNL--KNTPVGRGVLKAYFATIPSLP-ALVWVNLHAGGQVFKFPS------------EQSITEMNIISWLEKLKAGLEI-
T.g. 647 LNL--EKTPVGYGILRTYFSTTPPLP-VLLWVNFHAGGQVFKFPS------------EQSITEMNIISWLEKLQAGLEI-
X.t. 619 LNL--KNTPVGVGILQRYFGIVPSLP-QLVLIDFDSLGQVFSFPL------------DHHLTDVNIIYWLEMIKAGAEL-
D.r. 677 IHL--GRTPVGRSILEKYLGFVPPLP-SLVISQIKTGGEVFLFPS------------ERPLKAENIIRWIHDVEDRHEQ-
B.f. 687 MNMVS-PASLGAELLQFYV-PNAGLP-ALVMVK-HKTGGVFEYPY------------PDDLSKDKVTAWVRGCENGDIL-
S.k. 694 MEVGMNSNSMGSFILHSYID-EPSLP-TIVTVN-HDNGKLYNYPM------------GNDFSKQSISKWLQGISSGKAG-
S.p. 1139 MDISD-EESVGSQTLTTY-GFLPYSDQVTIAILDHALGEVCRCPY------------GNDPSETALTAWLEVSLESPKHC
C.i. 715 DG------KIIFTWMDSS----PNTP-GEYALNIHSGGKWEGAPIVFFDKKGSVMFRDVINAENKSAKWLTDCLAGKI--

```
                                 α                                                     α
H.s.       730 -HITILPAQEWKPPLPAYDFLSMIDAATSQRGTRKVPKCM---------------------------------KETDVQE
P.t.       730 -HITILPAQEWKPPLPAYDFLSMIDAATSHRGTRKVPKCM---------------------------------KETDVQE
M.mul.     736 -HITILPAQEWKPPLPAYDFLSMMDAATSQHSTRKIPKCM---------------------------------KETDVQE
C.f.       731 -HITILPAQEWKPPLPAYDFLSMMDAAASQHPTRKDPECT---------------------------------KETDVQE
B.t.       711 -PIKVLPAQEWKPPLPAYDFLSMMDAAASEHLARKVPECI---------------------------------KETDMHE
M.mus.     750 -PITVLSAQEWKPPLPAFDFLNMMDAPTSQAPTKKVLECQ---------------------------------KEAEVQE
R.n.       765 -PITVLSAQEWSPPLPAFDFLSMMDVPTSRAPTKKVLECQ---------------------------------KEAEVQE
G.g.       739 -PSSTLSEEDWKPPLPAYNFLQMMEAAGPEFTLHTSSH-----------------------------------QEDRREQ
T.g.       711 -PSSTLSEEDWKPPLPAYDFLQMMEPSVPELSPHPGDTP----------------------------------AQHPPKS
X.t.       683 -PVYSLSNEDWKPPLPDYNFLDVMDAEVPGFAAQKIRIQM---------------------------------KPNRRQK
D.r.       741 --------------------------------------------------------------------------------
B.f.       750 -ANNELQEEKWGPMMPGYDFLAFIDSEGDSAKQRNERSIH------------------------------H--DEADIET
S.k.       758 LQTGELKDGKWKPLLPAYDFLAMIDSEEAEYRKE-VEESMGGVFITDCICPSRPPKGLQHYKPQMRVPDKKSGKGKEKVN
S.p.      1205 SEARALIDREWKPLVPPYDFLKFMEDEEHSVGAGRRGFVL---------------------------------HPEEAAE
C.i.       780 EATYKLRDEEWKGRLPGYDLLQIMKDEGTYPVFPKKE-V-----------------------------------EEKQE

                                                *
H.s.       776 ---NDKEQHEDKSAVRKEP--IET--LRIKHWN----------RSNWFKEAEK---SFRRD-KELGCSKVN
P.t.       776 ---NDKEQHEDKSAVRKEP--IET--LRIKHWN----------RSNWFKEAEK---SFRRD-KEL------
M.mul.     782 ---NDKEQHEDKSAVRKEP--IET--LRIKHWN----------RSNWFKEAEK---SFRHD-KEL------
C.f.       777 ---NDKKQHEDKSAIIKEP--IET--LRIKHWN----------RSNWFKEGEK---SFRHDNKEL------
B.t.       757 ---SDKEQPDDKSAITEES--IGT--SKMKHSS----------RSSWLEEIKK---PLRHDNQEL------
M.mus.     796 ---SAELQPGDRSTARREP--VEM--LRIKRWN----------TANWPKDTQE---PFHHD-KEL------
R.n.       811 ---RAGQQPGDKSTTRREP--VEV--LRIKHWN----------RSNRPKESRE---PFHHD-KEL------
G.g.       783 Q---QLEGSEEKATVREEP--IEE--ARVKAEKAQGPGRDLRGTAPRLAGREK---QARRH-SEL------
T.g.       756 P---GGDPARGDPASREEP--SQQ--SAA--EEVGSPGRGLRGTAPRLAARDK---QGKRH-GEL------
X.t.       729 -VEDSPEGVEEESEAKKYP-PGSS--LRG--------------TVPKFIEQGK---KFRSH-TEL------
D.r.       741 --------PA-------------------------------------------------E--KGNS-----
B.f.       797 ----EGIEPEDEELPEEEEVPTEG--S-------------------RLSKPDV---KLHKH-TEL------
S.k.       837 GEEAQNEENEEV-IENTED--IDSSISDISLRQRK-------NKTEERKEQEDGDVQDEV--KNDN-----
S.p.      1252 ---GTDEKEEEK------P--EQD--SEDSEKV-----------DAAVKENQK---HSPKH-IEL------
C.i.       823 ---TVPR--G--------P--VHS--GKQKKVV-----------PQEPKE-EG---NQPKH-TEL------
```

Riemer et al., FIGURE S3
