# Supplementary material for: Identification of the PDI-Family Member ERp90 as an Interaction Partner of ERFAD
Source: PLoS One. 2011 Feb 16;6(2):e17037. doi: 10.1371/journal.pone.0017037 (PMC3040216; doi:10.1371/journal.pone.0017037)
Supplement: Text S1 — An overview of the cloning work performed to construct the pcDNA5FRT/myc-ERp90, pcDNA3.hygro/myc-ERp90, and pMAL-c2X/MBP-Xa-ERp90Trx3 plasmids. (DOC) [file pone.0017037.s005.doc]

**Supplementary Information, Riemer *et al.***

Primers and plasmid construction

| **Plasmids** | **Primers (5’-3’)** | **Restriction sites:** |
| --- | --- | --- |
| pcDNA3.hygro  myc-ERp90 | *Forward1:* GACAAGCTTCATGTTTTCCGGCTTCAATGTC  *Reverse1:* CCTCTTCAGAAATGAGCTTTTGCTCTGGTAAAGAGTTTACTGTTGGCATGTAAAAAATG  *Forward2:* CATGCCAACAGTAAACTCTTTACCAGAGCAAAAGCTCATTTCTGAAGAGG  *Reverse2:* CTGAGGACTCAGTTCTGGTAAAGAGTTAAGATCCTCCTCGGATATTAACTTCTG  *Forward3:* CATCCGCTCGAGTTACCAGAACTGAGTCCTCAGTCAGAAATATT  *Reverse3:* CATCCGCTCGAGTTAGTTCACTTTTGAGCATCCTAACTC | HindIII  Overlap extension with *Forward 2*  Overlap extension with *Reverse 1*  XhoI  XhoI  XhoI |
| pRSETminiT  His-ERp90Trx3 | *Forward:* GACGGATCCTTACCTTTGGAACTTACAGTGG  *Reverse:* CATGAATTCTCATGAAATCCTGTTGAGCTGG | BamHI  EcoRI |
| pMALc2X/MB-  Xa-ERp90Trx3 |  | BamHI & HindIII |

For pcDNA3.hygro/myc-ERp90 PCR reactions with the first (encoding the ERp90 signal sequence) and the second (encoding a myc_3_ tag) primer sets were performed. The purified PCR products were ligated in an overlap extension PCR using *Forward1* and *Reverse2*. This PCR product was cloned into pcDNA3.hygro and the resulting plasmid named pcDNA3.hygro/SP-myc_3_. A PCR amplifying mature ERp90 was then performed with the third primer set and ligated into pcDNA3.hygro/SP-myc_3_. The orientation of the insert was determined by restriction analysis. The final construct encodes a product where the myc_3_ tag is fused in-frame with mature ERp90 and inserted directly after the predicted ERp90 signal sequence.

For pRSETminiT/His-ERp90Trx3 a PCR reaction with the indicated primers was performed. The resulting product was digested with the indicated restriction enzymes and ligated into pRSETminiT.

For pMAL-c2X/MBP-Xa-ERp90Trx3, pRSETminiT/His-ERp90Trx3 was digested with BamHI and HindIII and the excised insert was ligated into pMAL-c2X (New England Biolabs).

The sequence of all plasmids was verified by DNA sequencing.

**References for Supplementary Information**

1. Edgar, R. C. (2004) MUSCLE: multiple sequence alignment with high accuracy and high throughput, *Nucleic Acids Res* *32*, 1792-1797.

2. Dong, G., Wearsch, P. A., Peaper, D. R., Cresswell, P., and Reinisch, K. M. (2009) Insights into MHC class I peptide loading from the structure of the tapasin-ERp57 thiol oxidoreductase heterodimer, *Immunity* *30*, 21-32.
